# Population Variability of Main Secondary Metabolites in *Hypericum lydium *Boiss. (Hypericaceae)

**Published:** 2015

**Authors:** Cüneyt Çirak, Jolita Radusiene, Liudas Ivanauskas, Valdas Jakstas, Necdet Çamaş

**Affiliations:** a *Ondokuz Mayis University, Vocational High School of Bafra, Samsun, Turkey.*; b *Nature Research Centre, Institute of Botany, Zaliuju Ezeru 49, Vilnius LT 08406, Lithuania.*; c *Medical Academy, Lithuanian University of Health Sciences, A. Mickeviciaus 9, Kaunas LT 44307, Lithuania.*

**Keywords:** Chemical diversity, Flavonoids, Hyperforins, Hypericins, *Hypericum lydium*

## Abstract

In the present study, we investigated the variation in the content of naphthodianthrones hypericin and pseudohypericin, phloroglucinol derivatives hyperforin and adhyperforin, the phenolic acids as chlorogenic acid, neochlorogenic acid, 2,4-dihydroxybenzoic acid, and the flavonols, namely, hyperoside, isoquercitrin, quercitrin, quercetin, avicularin, rutin, (+)-catechin and (-)-epicatechin, and biflavonoid amentoflavone among wild *H. lydium* Boiss. populations from five different growing sites of Turkey for the first time. The aerial parts representing a total of 30 individuals were collected at full flowering and dissected into floral, leaf and stem tissues. After dried at room temperature, the plant materials were assayed for chemical contents by HPLC. The populations varied significantly in the content of chemical compounds. Among different plant parts, flowers were found to be main repository site of hyperforin, adhyperforin, hypericin, pseudohypericin, amentoflavone, quercetin, avicularin, rutin and (+)-catechin accumulations whereas rest of the compounds tested were accumulated primarily in leaves in all growing localities. The stems were the least accumulative organ that did not yield hyperforin, adhyperforin and rutin. The chemical diversity among the populations and plant parts is discussed as being possibly the result of different environmental, morphological and genetic factors.

## Introduction

**Figure 1:**
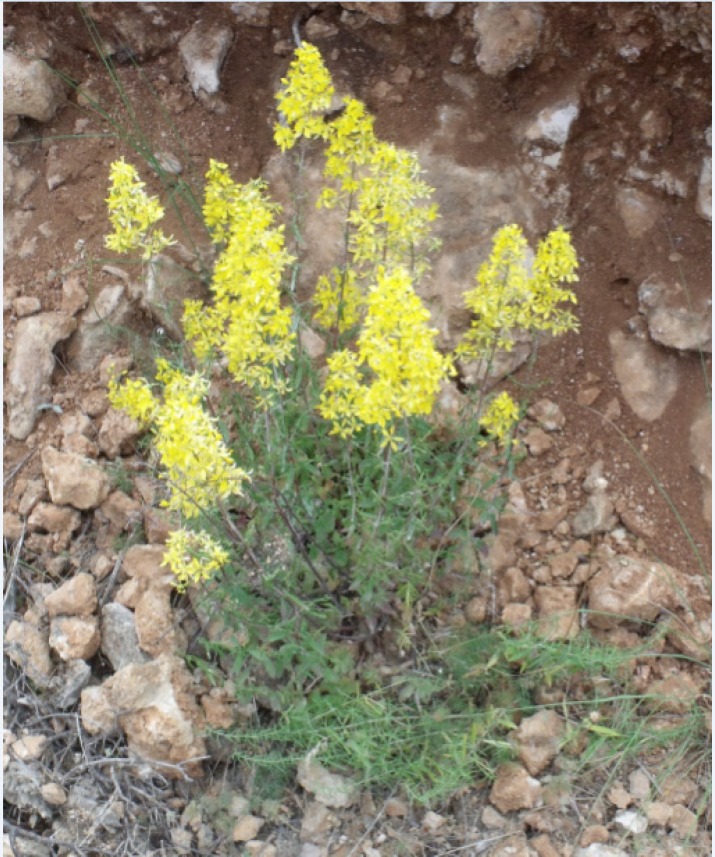
A view from *H. lydium *plant at flowering in its native habitat.

The genus *Hypericum* L. (Hypericaceae) includes 484 species that occur naturally in every continent of the world, except Antarctica (1). *Hypericum *genus are well known for their use in traditional medicine due to their various medicinal properties (2). In particular, extracts of *H. perforatum *L., the most abundant and well known species, are now widely used in Europe as a drug for the treatment of depression (3). The genus is represented in the flora of Turkey by 89 species from 19 sections and 43 species are reported to be endemic (4). *H. lydium *Boiss. (Hypericaceae) is a herbaceous perennial establishing rare populations on stony slopes in *Pinus* copse. Plant shoots up to 60 cm in length with yellow inflorescence in pyramidal shape and typical dark glands on all aerial parts (5) (Figure 1). The plant is known as “sancı otu and mayasılotu” in Turkish folk medicine. It has traditionally been used in the treatments of menstrual disorders and stomach pains as infusion (6). Besides, *H. lydium* is a well known wound healer and its decoction is also used as tea internally in treatment of hemorrhoids (7, 8). Results from recent studies reporting the antimycobacterial (9) and antioxidant (10) properties of *H. lydium* sign out the great potential of this species as a promising medicinal plant.

Naphthodianthrones, phloroglucinol derivatives, essential oils and several phenolics are thought to be main bioactive compounds in *Hypericum* extracts which possess a broad array of biological activity (11). Among the chemicals, hypericins and hyperforins are considered to be synergistically responsible for the antidepressant activity of *Hypericum* extracts (12). Hyperforin also exhibits anti-inflammatory (13), antitumoral (14) and antiangiogenic (15) effects. Hypericins have been reported to exhibit antiviral, antiretroviral, photodynamic, antibacterial, antidepressant and antitumoral activities (16). Although hyperforin and hypericins have been reported to mainly contribute to the pharmacological effects of *Hypericum* extracts, flavonoids and phenolic acids have also made an important contribution to the antidepressant (17) and antimicrobial (18) activities. 

Growing market niche for *Hyperici herba* has resulted in excessive amount of investigations on the pharmacological activities of *Hypericum* genus. This is especially true for *H. perforatum*, which is the most prevalent and deeply studied species. Although many of studies have been undertaken on the chemical profile of *H. perforatum*, a relatively small number of other *Hypericum* species have been studied for the presence of new compounds so far. In previous studies, *H. lydium* was reported to contain chlorogenic acid, rutin, hyperoside, apigenin-7-O-glucoside, quercitrin, quercetin, hypericin and pseudohypericin (19, 20). However, population variability of the chemical compounds has not yet been studied for this species. Thus, in the present study we investigated the variation in the content of naphthodianthrones hypericin and pseudohypericin, phloroglucinol derivatives hyperforin and adhyperforin, the phenolic acids chlorogenic acid, neochlorogenic acid, and 2,4-dihydroxybenzoic acid, the flavonols hyperoside, isoquercitrin, quercitrin, quercetin, avicularin, rutin, (+)-catechin and (-)-epicatechin, and biflavonoid amentoflavone among five different populations of *H. lydium* from Turkey for the first time. Besides, hyperforin, adhyperforin, neochlorogenic acid, 2,4-dihydroxybenzoic acid, amentoflavone, isoquercitrin, avicularin, (+)-catechin and (-)-epicatechin were not detected previously in this species. Here, we also describe the first occurrence of the corresponding compounds in *H. lydium*. 

## Experimental

*Plant material*

The plant material was described in our previous study (20). The species were identified by Dr. Samim Kayikci, Mustafa Kemal University, Faculty of Arts and Sciences, Department of Biology, Turkey. Voucher specimens were deposited in the herbarium of Ondokuz Mayis University Agricultural Faculty and the numbers of the voucher specimens are given in Table 1. 

*Experimental procedures*

The aerial parts of *H. lydium *plants representing a total of 30 shoots were collected at full flowering stage from five sites of Turkey (Table 1). The top of 2/3 plants was harvested between 11:00 am and 13:00 pm. Conditions on the day of collection were clear and sunny at all sites. Temperatures ranged from 26 to 32 °C. After collection, 10 individuals were kept as whole plants and the rest were dissected into floral, leaf and stem tissues. The plant materials were dried at room temperature (20 ± 2 °C), and subsequently assayed for chemical contents by HPLC.

**Table 1:** Geographical data and seasonal climatic conditions of *Hypericum lydium *growing localities from Turkey.

**Sites**	**Collection date**	**Voucher no.**	**Latitude (N)**	**Longitude (E)**	**Elevation (m)**	**Mean temperature (°C)**	**Precipitation (mm)**	**Habitat**
Havza	June 14, 2012	BMYO # 9/1	40 51΄	35 29΄	850	10.01	850	Rocky and open slopes
Pozantı	June 18, 2012	BMYO # 9/2	34 54΄	37 28΄	1074	14.10	535	Arid pasturelands
Gümüş	June 19, 2012	BMYO # 9/3	40 52΄	35 14΄	1200	11.00	825	Igneous slopes and rock ledges
Gaziantep	June 14, 2012	BMYO # 9/4	34 54΄	37 28΄	795	15.17	525	Arid pasturelands
Bolu	June 11, 2012	BMYO # 5/5	41 04΄	20 06΄	1150	11.12	819	Arid pasturelands

*Preparation of plant extracts*

Air-dried plant material was mechanically ground with a laboratory mill to obtain a homogenous drug powder. Samples of about 0.1 g (weighed with 0.0001 g precision) were extracted in 10 ml of 100% methanol by ultrasonication at 40 ºC for 30 min. in a Sonorex Super model RK 225H ultrasonic bath. The prepared extracts were filtered through a membrane filter with pore size of 0.22 µm (Carl Roth GmbH, Karlsruhe, Germany) and kept in a refrigerator until analysis. 

The extracts for naphthodianthrones analysis were exposure to light under xenon lamp (765 W/m^2^) for 8 min. due to the photoconversion of protohypericins into hypericins.

*HPLC analysis and quantification*

A Waters Alliance 2695 (Waters, Milford, USA) separation module system equipped with Waters 2487 UV/V is and Waters 996 PDA diode-array detectors, was used for HPLC analysis. Data were analyzed using Empover Software chromatographic manager system (Waters Corporation, Milford, USA).

Separation of flavonoids, epicatechin and hyperforin was carried out on SunFire C18 column (3.5 μm, 150 mm × 3.0 mm *i.d*.; Waters, Milford, USA) with 10 mm guard-precolumn. The binary gradient elution method was used for detection of corresponding compounds. The mobile phase consisted of water Milli-Q acidified with 0.3% phosphoric acid as eluent A and acetonitrile containing 0.3% phosphoric acid as eluent B. The elution profile was used as follows: 0-12 min 16% B, 12-18 min (B 16→53%), 18-18.1 min (B 53→97%), 18.1-29 min (B 97→97%), 29-30 min (B 97→16%). Flow rate was 0.6 mL min^-1^ at a constant 25 ^o^C column temperature. The volume of extract injected was 10 µL. Peaks were detected at a wavelength range of 270-360 nm. 

The ACE C18 column (5.0 μm, 250 × 4.6 mm *i.d*.; MAC-MOD Analytical, Inc) with guard-precolumn was used for separation of phenolic acids, catechin and hypericins. 

The mobile phase of gradient elution of phenolic acids and catechin was composed of eluent A: water acidified with 0.5% glacial acetic acid, and eluent B: acetonitrile. The separation was performed using the following program: 0-30 min (B 5→35%), 30-36 min (B 35→90%), and 36-37 min (B 90→5%). The flow rate was 1.0 mL min^-1 ^at 25^o^C column temperature. Peaks were detected at a wavelength range 277-324 nm.

Naphthodiantrones were analysed according to the modified European Pharmacopoeia method (21). The mobile phase of isocratic elution of hypericin and pseudohypericin consisted of ethyl acetate, aqueous 0.1 M sodium dihydrogen phosphate solution, adjusted to pH 2.0 using phosphoric acid and methanol (16:17:67% v/v). The flow rate was 1.0 mL min^-1^; volume of extract injected - 20 µL. Detection was performed at 590 nm wave length at 40 ^o^C column temperature.

Chromatographic peaks were identified by comparing retention times of samples with those of the reference standards. Furthermore, in order to confirm the identity of the eluted constituents, spectral characteristics of the eluting peaks were recorded with diode-array detector and compared with UV spectra’s of authentic standards.

Quantification of compounds was carried out by the external standard method. Standards stock solutions were prepared freshly in methanol and diluted in appropriate quantities to obtain a set of corresponding concentration ranges for the study of linearity. A calibration curve for each of the compounds was constructed by plotting peak areas versus the respective compound concentration and calculated by linear regression analysis. The regression coefficients (r^2^ ≥ 0.999) of all calibration curves indicated that, in the ranges of standard concentrations analyzed, the peak areas were directly proportional to the concentrations and, thus, methods presented adequate linearity. The precision of the method was demonstrated for all analytes, since all the obtained relative standard deviations (RSD) values were lower than 5.0%. The retention time, linear range, regression equation, correlation coefficient and RSD values of each analysis are summarized in Table 2. The concentration of compounds was expressed as mg/g dry mass (DM). Solvents used were HPLC grade and purchased from Roth GmbH (Karlsruhe, Germany). Water was filtered through the Millipore HPLC grade water preparation cartridge (Millipore, Bedford, USA). Reference substances were purchased from ChromaDex (Santa Ana, USA), Sigma-Aldrich (Saint Louis, USA) and HWI ANALYTIK GmbH (Germany).

**Table 2:** The retention time, linear range, regression equation and correlation coefficient, precision of each detected analytes of HPLC analysis on examined populations of *Hypericum lydium*

**Analytes**	**Retention time, min**	**Linearity range (μg/mL)**	**R** ^2^	**Regression equation**	**Precision, RSD (%)**
2,4-Dihydroxybenzoic acid	13.7	0.31–19.60	0.99977	Y = 1.94·10^4^ X + 3.34·10^3^	0.28
Neochlorogenic acid	15.0	0.61–196.00	0.9999	Y = 3.43·10^4^X - 4.83·10^3^	0.82
Chlorogenic acid	21.5	0.30–194.00	0.9999	Y = 3.05·10^4^ X + 3.79·10^3^	0.36
Caffeic acid	24.5	0.31–19.60	0.9999	Y = 5.25·10^4^X + 7.00·10^3^	0.18
Rutin	10.1	0.14–90.95	0.9999	Y = 2.77·10^4^X - 5.18·10^3^	1.02
(-)-Epicatechin	4.5	0.15–194.00	0.9999	Y = 1.08·10^4^X + 1.46·10^3^	1.36
Hyperoside	11.9	0.16–99.00	0.9999	Y = 5.27·10^4^X + 3.24·10^2^	0.52
Isoquercetin	12.8	0.16–99.00	0.9999	Y = 4.46e·10^4^X - 3.24·10^3^	0.66
Avicularin	17.0	0.15–19.16	0.9997	Y = 3.44·10^4^X - 1.73·10^3^	2.83
Quercitrin	17.2	0.15–98.00	0.9999	Y = 3.23·10^4^X - 1.37·10^3^	0.31
Quercetin	19.3	0.15–190.00	0.9996	Y = 3.52·10^4^X + 4.18·10^4^	4.60
(+)-Catechin	19.7	0.30–95.00	0.9997	Y = 1.20·10^4^X + 3.85·10^3^	3.19
Amentoflavone	20.1	0.14–179.94	0.9999	Y = 3.48·10^4^X + 1.27·10^4^	1.34
Hyperforin	23.3	3.11–199.00	0.9999	Y = 2.42·10^4^ X + 6.04·10^3^	0.78
Adhyperforin	26.0	1.02–65.00	0.9999	Y = 2.42·10^4^ X + 1.86·10^3^	0.40
Pseudohypericin	3.4	0.38–96.20	0.9998	Y = 6.84·10^4^ X + 1.13·10^4^	2.06
Hypericin	9.4	0.37–95.10	0.9997	Y = 1.00·10^5 ^X + 2.25·10^4^	2.45

*Data analysis*

Data for all contents of plant material including whole plant, stem, leaf and flower were objected to ANOVA and significant differences among mean values were tested with the Duncan Multiple Range Test (P < 0.01) by using MSTAT-C statistical software (Russell D. Freed, Crop and Soil Sciences Department, Michigan State University). Mean values of the chemical contents were normalized using $X^{,}=\sqrt[{2}]{x}+1$ transformation before conducting ANOVA, when necessary, because some chemicals were not detected in several cases.

## Results

Hypericin, pseudohypericin, hyperforin, adhyperforin, chlorogenic acid, neochlorogenic acid, 2,4-dihydroxybenzoic acid, amentoflavone, hyperoside, isoquercitrin, quercitrin, quercetin, avicularin, rutin, (+)-catechin and (-)-epicatechin contents in plant materials including whole shoots, stem, leaf and flower tissues varied significantly with populations (P < 0.01). Hyperforin and adhyperforin were detected only in plants from Havza and Pozantı populations and content of the both compounds was significantly higher in whole shoots of Pozantı population (6.78 and 5.12 mg g^-1^ DM, respectively). The highest level of amentoflavone and isoquercitrin accumulations was also observed in whole shoots of the same population (2.98 and 3.92 mg g^-1^ DM, respectively). Hypericin and pseudohypericin contents in whole shoots were similar among populations while plants from Gaziantep population produced the highest amount of both hypericin forms (1.02 and 1.12 mg g^-1^ DM hypericin and pseudohypericin, respectively). Similarly, quercitrin, rutin and (-)-epicatechin contents were evidently higher in whole shoots of this population when compared to those of other populations which accumulated the corresponding compounds at moderate level (37.78, 0.78, 13.28 mg g^-1^ DM, respectively). Chlorogenic, neochlorogenic and 2,4-dihydroxybenzoic acids and avicularin contents of whole shoots reached the highest level in Bolu population (2.01, 9.97, 0.16 and 0.77 mg g^-1^ DM, respectively). Generally lower accumulation levels were observed in Gümüş and Havza populations for all tested compounds with the exception of quercetin, the highest level of which was produced by plants from Havza population (1.52 mg g^-1^ DM) and hyperoside, (+)-catechin which were accumulated by plants from Gümüş population at the highest level (8.12 and 0.36 mg g^-1^ DM, respectively) (Table 3).

**Table 3:** Hypericin (1), pseudohypericin (2), hyperforin (3), adhyperforin (4), chlorogenic acid (5), neochlorogenic acid (6), 2,4-dihydroxybenzoic acid (7), amentoflavone (8), hyperoside (9), isoquercitrin (10), quercitrin (11), quercetin (12), avicularin (13), rutin (14), (+)-catechin (15) and (-)-epicatechin (16) contents (mg g^-1^ DM) in *H. lydium *whole shoots from wild populations located in Turkey

**Populations**	**Compounds **															
	**1**	**2**	**3**	**4**	**5**	**6**	**7**	**8**	**9**	**10**	**11**	**12**	**13**	**14**	**15**	**16**
Havza	0,15 c^a^	0,37 b	0,07	0,03	0,12 b	7.78 b	0.01 c	2.93 a	6.27 b	3.77 a	0,04 c	1.52 a	0.04 b	0.12 c	0.25 b	4.52 b
Pozantı	0,04 d	0,23 c	6,78	5,12	0,19 b	4.36 c	0.01 c	2.98 a	6.13 b	3.92 a	0.08 c	0.27 c	0.02 b	0.23 b	0.29 b	3.97 b
Gümüş	0,25 b	0,15 c	0	0	0,03 c	7.26 b	0.01 c	1.15 b	8.12 a	3.85 a	0,09 c	0.32 c	0.04 b	0.25 b	0.36 a	4.27 b
Gaziantep	1,02 a	1,12 a	0	0	0,01 c	2.27 d	0.09 b	0.87 c	0.85 d	0.22 c	37.78 a	0.26 c	0.04 b	0.78 a	0.24 b	13.28 a
Bolu	0,02 d	0,20 c	0	0	2,01 a	9.97 a	0.16 a	0.82 c	3.27 c	3.77 b	1.96 b	0.98 b	0.77 a	0.11 c	0.23 b	2.98 b

a Values followed by different small letters in each column are significantly different (P < 0.01) according to Duncan Multiple Range test.

**Figure 2:**
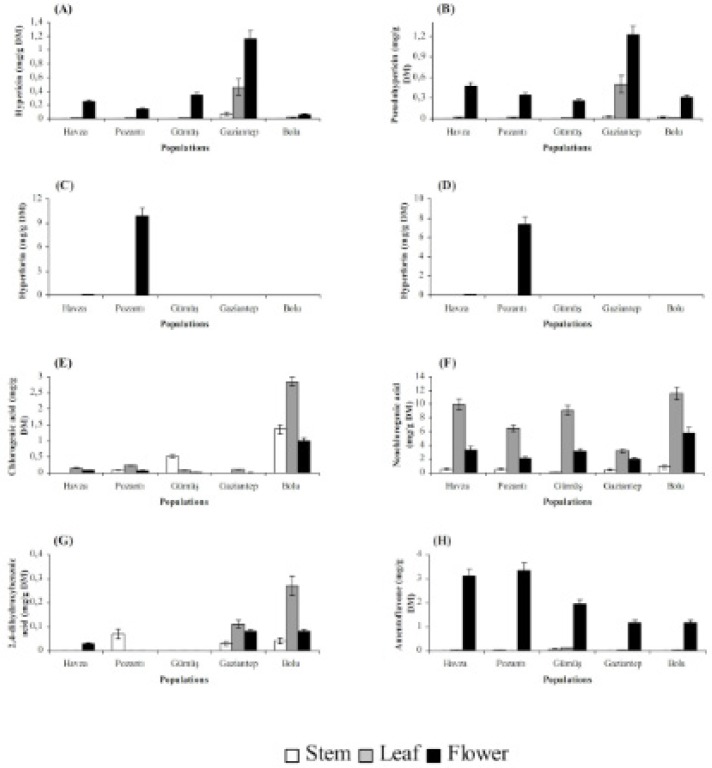
Hypericin (A), pseudohypericin (B), hyperforin (C), adhyperforin (D), chlorogenic acid (E), neochlorogenic acid (F), 2,4-dihydroxybenzoic acid (G) and amentoflavone (H) contents in stem, leaf and flower of *H. lydium* species from wild populations located at Turkey (Bars are ± s.e).

**Figure 3:**
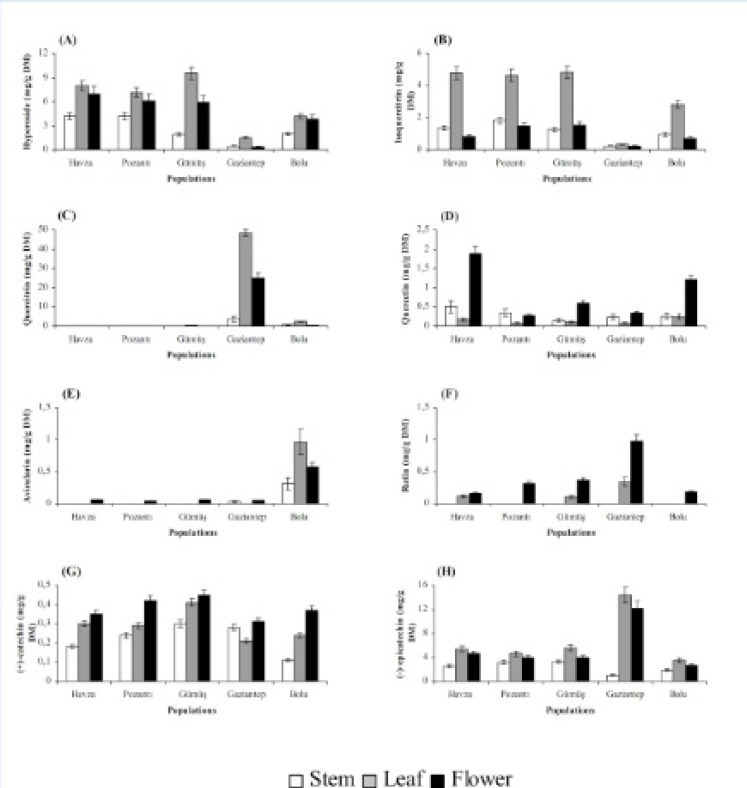
Hyperoside (A), isoquercitrin (B), quercitrin (C), quercetin (D), avicularin (E), rutin (F), (+)-catechin (G) and (-)-epicatechin (H) contents in stem, leaf and flower of *H. lydium* species from wild populations located at Turkey (Bars are ± s.e).

Stems, leaves and flowers also differed significantly (P < 0.01) with respect to chemical accumulation (Figures 2 and 3). The highest content of hyperforin, adhyperforin, hypericin, pseudohypericin, amentoflavone, quercetin, avicularin, rutin and (+)-catechin was determined in flowers whereas rest of the compounds tested were accumulated primarily in leaves in all growing localities. Stems were the least accumulative organ and they did not yield hyperforin, adhyperforin and rutin.

## Discussion

Pharmacological activity of *Hypericum* extracts are reported to be influenced significantly by the instability of chemical compounds level in *Hypericum* genus of different origins (22). Thus, many of efforts have been dedicated to the population variability of phytochemicals in different *Hypericum* species especially over the last decade. Accumulation levels of hypericin and pseudohypericin in *H. perforatum* from different growing regions of Switzerland (23), USA (24), Australia (25) as well as Turkey (26) and Lithuania (27) were investigated. Quercitrin, quercetin, rutin, hyperoside, chlorogenic acid (26) and hyperforin (28) content variations were also demonstrated among different populations of the same species growing wild in the other regions of the world. Regarding the changes of chemical levels observed in wild populations of other *Hypericum *species, several reports are available in the current literature. Content of volatile constituents varied markedly depending on the geographic origin of wild plants in *H. perfoliatum* L.*, H. humifusum* L.*, H. linarifolium *Wahl. and* H. pulchrum* L. (29). Hypericin, pseudohypericin, rutin, hyperforin, hyperoside, apigenin-7-O-glucoside, quercitrin, quercetin, kaempferol and amentoflavone contents were substantially different among *H. triquetrifolium* Turra from four regions of Turkey (30, 31). Hypericin, pseudohypericin, hyperforin, hyperoside, isoquercetin, chlorogenic acid, quercitrin, rutin, and quercetin were accumulated in significantly different amounts in wild *H. orientale *L. from 10 wild populations of Northern Turkey (32). 

Results from the above mentioned studies pointed out the effect of geographic origin as a significant factor influencing the chemical instability observed among wild *Hypericum* genus. In the present study, similarly, significant differences were detected in accumulation levels of the secondary metabolites tested among *H. lydium* from five different origins. The investigated populations were located in different sites of North and South Turkey which varied by some climatic and geographic factors that form different growing conditions as seen in Table 1. The huge variation observed in chemical contents among the populations could be partly attributed to phenotypic plasticity of plants to adapt to different environmental conditions. On the other hand, the genetic variation among plants may have an impact on differences in plant chemistry. For example, Havza and Pozantı populations are separated by a distance of 550 km. and have apparently different growing conditions. Therefore, they should typify explicit populations. However, both populations yielded similar amount of chlorogenic acid, 2,4-dihydroxybenzoic acid, amentoflavone, hyperoside, isoquercitrin, quercitrin, avicularin, (+)-catechin and (-)-epicatechin. On the contrary, (-)-epicatechin and quercitrin accumulations were tremendously higher in plants from Gaziantep population when compared to those of other geographically distant or close populations. However, it is noteworthy to note that elaborative analyses should be carried out by using molecular and biochemical markers to clarify the genetic multiplicity which is the most reliable way to distinguish definitely the effects of genetic and environmental factors on the observed phytochemical variation among wild populations. For example, Afef *et al.* (33) assessed the genetic variation within and among seven *H. humifusum* populations from different growing localities of Tunisia by using 166 RAPD markers and 11 isozymic polymorphic loci. They reported a high genetic diversity among the populations and signed out the essentiality for further researches on the variability in chemical content/composition among wild populations as well as its connection with genetic instability. Multiple molecular, phytochemical and morphological studies have currently been undertaken to illuminate the variations within and among species of *Hypericum* genus (34).

The localization of the secretory structures in which bioactive substances are synthesized varies greatly among plant tissues, and for that reason, the levels of phytochemicals in a particular *Hypericum *tissue depend on the relative abundance of these secretory structures (35). As a result, organ-dependence of a given chemical is common among *Hypericum *species. In the present study, flowers were found to be superior over leaves for hyperforin, adhyperforin, hypericin, pseudohypericin, amentoflavone, quercetin, avicularin, rutin and (+)-catechin accumulations while chlorogenic acid, neochlorogenic acid, 2,4-dihydroxybenzoic acid, hyperoside, isoquercitrin, quercitrin and (-)-epicatechin were accumulated mainly in leaves. Similar to our results, it was found that flowers of *H. perforatum* accumulated larger amount of hypericin, hyperforin, rutin and quercetin while its leaves had the highest level of hyperoside, isoquercitrin and chlorogenic acid (36, 27). The same trend for compound accumulation was observed in *H. origanifolium* Willd., and *H. perfoliatum* (37, 38) as well as in *H. aviculariifolium *subsp. *depilatum *var. *depilatum* (Freyn and Bornm.) Robson var.* depilatum* and *H. orientale *L. (39). 

According to taxonomical classification, *H. lydium *belongs to the section *Drosanthe* Spach (5). To the best of our knowledge, only one species of this section, *H. scabrum *L., had been subjected to detailed analyses for secondary metabolite contents (40). The comparison of chemical composition of these species revealed that the two members of *Drosanthe* section have alike chemical profile which include hypericin, pseudohypericin, chlorogenic acid, rutin, hyperoside, quercitrin, quercetin and amentoflavone. Among the chemicals, hypericin and pseudohypericin have a distinct taxonomic importance for the infrageneric classification of the genus *Hypericum* (1) since corresponding compounds were reported to be specific only for the taxa of phylogenetically more advanced sections of *Hypericum* genus (41). Hence, detection of hypericins as well as the other chemicals examined in *H*.* lydium *in the present study consolidates the taxonomic position of the section* Drosanthe* within the genus *Hypericum*. 

## Conclusions

Te present results indicate a remarkable diversity in the phytochemical contents of *H. lydium *from different growing localities of Turkey. Geographical distribution of this medicinal herb may be a significant reason of chemical instability and should be regarded to avoid the huge variability in secondary metabolite profile of standardized *Hypericum* extracts. Further molecular, morphological and chemical studies on the relationship between genetic and chemical profiles of wild populations are needed to make more constitutional inferences. In the present study, we also describe the first occurrence of hyperforin, adhyperforin, neochlorogenic acid, caffeic acid, 2,4-dihydroxybenzoic acid, amentoflavone, isoquercitrin, avicularin, (+)-catechin (16) and (-)-epicatechin in this species. The present data could also be helpful in explanation of the chemotaxonomical significance of the corresponding compounds as well as phytochemical evaluation of *H. lydium.*
